# A High-Precision Ionospheric Channel Estimation Method Based on Oblique Projection and Double-Space Decomposition

**DOI:** 10.3390/s25185727

**Published:** 2025-09-14

**Authors:** Zhengkai Wei, Baiyang Guo, Zhihui Li, Qingsong Zhou

**Affiliations:** College of Electronic Engineering, National University of Defense Technology, Hefei 230037, China; weizhengkai19@nudt.edu.cn (Z.W.); guobaiyang@nudt.edu.cn (B.G.); lizhihui_16@163.com (Z.L.)

**Keywords:** ionospheric channel estimation, high-precision delay estimation, CC-OPMP, dual-space decomposition, anti-interference correlation metric

## Abstract

Accurate ionospheric channel estimation is of great significance for acquisition of ionospheric structure, error correction of remote sensing data, high-precision Synthetic Aperture Radar (SAR) imaging, over-the-horizon (OTH) detection, and the establishment of stable communication links. Traditional super-resolution channel estimation algorithms face challenges in terms of multipath correlation and noise interference when estimating ionospheric channel information. Meanwhile, some super-resolution algorithms struggle to meet the requirements of real-time measurement due to their high computational complexity. In this paper, we propose the Cross-correlation Oblique Projection Pursuit (CC-OPMP) algorithm, which constructs an atom selection strategy for anti-interference correlation metric and a dual-space multipath separation mechanism based on a greedy framework to effectively suppress noise and separate neighboring multipath components. Simulations demonstrate that the CC-OPMP algorithm outperforms other algorithms in both channel estimation accuracy and computational efficiency.

## 1. Introduction

**Figure 1:**
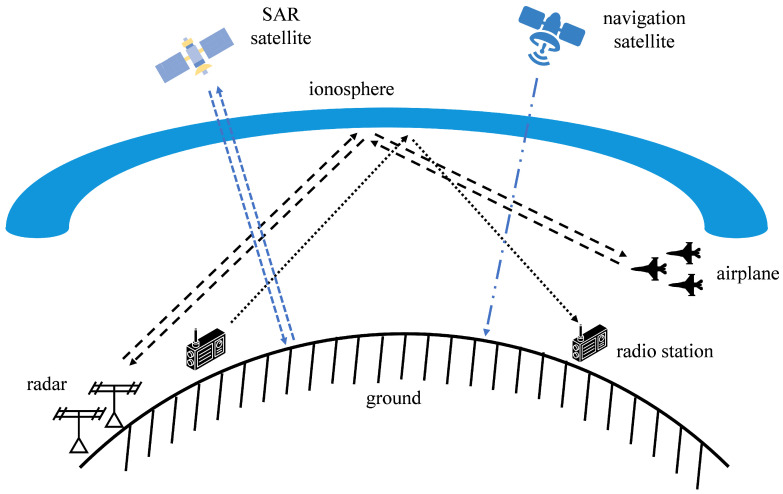
Ionospheric channel in multi-system operations.

The ionosphere is a plasma region in the Earth’s upper atmosphere. Its physical properties directly affect the propagation laws of electromagnetic waves. As shown in Figure 1, for satellite-based navigation satellites, the signals will experience delays and path deviations when passing through the ionosphere, resulting in a decline in navigation accuracy [1,2]. For SAR imaging, the ionosphere is the key medium through which SAR signals pass. The ionosphere causes delays, distance shifts, and phase distortions in SAR signals, which leads to SAR image distortion and the appearance of artifacts [3]. OTH radar utilizes the reflection characteristics of the ionosphere to achieve ultra-long-distance detection [2,4]. The properties of the ionosphere affect the stability of the signal propagation path, which determines the radar positioning accuracy. Meanwhile, the multipath echoes generated by reflections at different altitudes will form false targets, increasing the difficulty of target recognition. Communicating through the reflection characteristics of the ionosphere is the main method of shortwave communication [5,6]. The structure and changing patterns of the ionosphere have a significant impact on the stability of shortwave communication systems. Accurately obtaining the ionospheric channel state information (CSI) is the core key to solving the problem of the ionosphere’s impact on signal transmission in various systems, promoting more accurate satellite navigation, clearer SAR imaging, more reliable radar detection, and more stable shortwave communication [7].

Accurately detecting the channel information of the ionosphere and inverting the structural characteristics of the ionosphere can effectively eliminate signal distortion and significantly improve the geometric accuracy of remote sensing [8,9]. C. C. Watson proposed the Watson model to describe the ionospheric propagation characteristics of narrowband signals. A global ionosphere model based on spherical harmonic functions was proposed in 1988, which constructed the ionosphere electron total content (TEC) map by expanding in spherical harmonic coefficients. The IRI-2020 developed by the Space Research Council and the International Radio Science Union is currently widely used in ionospheric research and radio communication [10,11]. Accurate estimation of ionospheric channel parameters is of great significance for improving the precision of navigation systems, the accuracy of SAR imaging, the stability of shortwave communication links, and the detection accuracy of OTH radars. Additionally, the channel estimation algorithm also acquires the ionospheric CSI. Traditional channel estimation methods, such as the cross-correlation (CC) [12], can achieve delay estimation under ideal conditions. However, their resolution is fundamentally constrained by the Rayleigh criterion. This limitation causes partial multipath components to alias, making effective separation difficult [13,14]. In practical remote sensing scenarios, signals reflected or scattered by mountains, urban structures, or the sea surface arrive at the receiver via a finite number of paths, resulting in sparse multipath channel characteristics [15,16,17]. Super-resolution algorithms based on compressive sensing leverage this sparsity property for widespread application in channel estimation. Among these, matching pursuit (MP) [18], orthogonal matching pursuit (OMP) [19], sparsity adaptive matching pursuit (SAMP) [20,21], regularized orthogonal matching pursuit (ROMP) [22,23,24], and compressive sampling matching pursuit (CoSaMP) [25] represent classical greedy algorithms. These methods iteratively select the atom exhibiting maximum correlation with the residual signal to construct the support set for signal representation. The observed signal is subsequently reconstructed through a linear combination of atoms within the support set, approximating a local optimal solution. While demonstrating strong performance for super-resolution time delay estimation as classical compressive sensing algorithms, greedy approaches still exhibit limitations in practical multipath scenarios. Under the influence of low SNR and strong multipath correlation, greedy algorithms suffer from time delay estimation deviations, constraining estimation accuracy [26,27,28].

Recent research indicates that soft-thresholding-based greedy algorithms [29] and sparse Bayesian learning (SBL) techniques [30,31,32] partially alleviate the adverse effects of noise and signal correlation. However, both algorithms suffer from high computation complexity. The soft-thresholding approach relies on repeated threshold adjustments and iterations. SBL involves extensive posterior probability calculations and matrix inversions, causing computational complexity to increase dramatically with data scale. These characteristics make it challenging to balance accuracy and computational complexity in practical implementations [29,33,34]. Reference [35] proposes a low-complexity sparse adaptive channel estimation scheme based on dynamic thresholds, which significantly improves computational efficiency and estimation accuracy.

In multipath delay estimation, the strong correlation between multipath components often leads to significant delay estimation bias in traditional greedy algorithms and severely limits multipath resolution capability. To address this challenge, this paper proposes a new algorithm named Cross-correlation Oblique Projection Matching Pursuit (CC-OPMP), which significantly improves the resolution and anti-interference performance of multipath delay estimation while maintaining low computational complexity. Compared with traditional sparse greedy methods, the core innovations of CC-OPMP are reflected in the following two aspects.

Firstly, this paper designs a dual-space decomposition mechanism based on oblique projection. This mechanism decomposes the received signal space into a signal subspace and its orthogonal complement subspace, and then uses oblique projection technology to accurately project multipath components into their corresponding subspaces. This process effectively eliminates mutual interference between multipaths, overcomes the problem of reduced resolution of traditional greedy algorithms in strong-correlation multipath environments, and significantly improves the accuracy of delay estimation.

Secondly, CC-OPMP innovatively conducts the entire estimation process in the correlation domain and designs a new correlation measurement criterion. By leveraging the statistical irrelevance between signals and noise in the correlation domain, the algorithm significantly improves the output SNR, enabling it to maintain excellent estimation robustness even under low SNR conditions. At the same time, CC-OPMP maintains a linear computational complexity comparable to that of the classic OMP algorithm. Therefore, it features both high resolution and high real-time performance, making it suitable for real-time processing requirements in practical systems.

## 2. System Model

**Figure 2:**
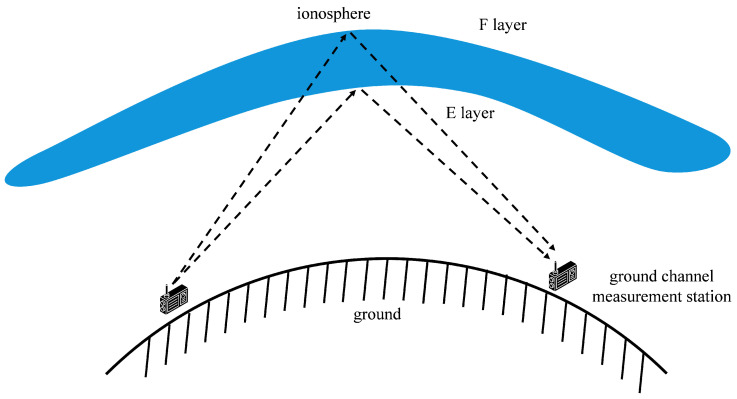
Ionosphere channel measurement system.

The ionosphere is a plasma region with a certain thickness in the Earth’s atmosphere and it contains different layered structures such as the E-layer and F-layer. When radio signals are directed towards the ionosphere, due to the refraction and reflection effects of charged particles in the ionosphere, the signals do not propagate along a single path. Instead, they are reflected in different layers and reach the receiving end through multiple paths. The core working principle of ionospheric channel measurement system is as follows: high-frequency signals are transmitted by ground stations, and reflected by the ionosphere. The system receives and analyzes the reflected signals, extracting key parameters such as time delay and amplitude to obtain the channel state information of the ionosphere. The channel measurement system is shown in Figure 2.

The ground station transmits high-frequency signals, which will be reflected at different heights in the ionosphere. The reflection positions are mainly concentrated in the E layer at 90–150 km and the F layer at approximately 150–800 km and the signal echoes return to the ground via multiple paths. The ionosphere system response function can be modeled as

(1) $$h\left(t\right)=\sum_{i=1}^{L}\alpha_{i}\delta \left(t-\tau_{i}\right)$$

where h(t) represent system response function, $\tau_{i}$ and $\alpha_{i}$ represent the delay and attenuation coefficients of the different path components of the multipath signal, respectively, and *L* represents the total number of paths.

In a single-input–single-output (SISO) channel measurement system, the signal passes through reflection, direct, and scattering during propagation, and reaches the receiving end through different paths. Each path has a different signal delay. The multipath signal model can be expressed as [36]

(2) $$y\left(n\right)=\sum_{i=1}^{L}\alpha_{i}x\left(n-\tau_{i}\right)+w\left(n\right),n=1,2,\ldots ,N\;$$

where y(n) represents the received signal, x(n) represents the transmitted signal, w(n) represents additive white Gaussian noise, *N* represents the number of sampling points.

Assume that the signal and noise are not correlated, while the signals are correlated. To enhance the system’s robustness against noise, the received signal is correlated with the transmitted signal to construct the multipath signal model after correlation $r_{xy}\left(\tau \right)$ [37]

(3) $$\begin{array}{cc} r_{xy}\left(\tau \right) & =E[y(n)*x(n+\tau )]\\ \; & =\sum_{i=1}^{L}\alpha_{i}r_{xx}\left(\tau -\tau_{i}\right)+r_{xn}\left(\tau \right)\\ \; & =r_{xx}\left(\tau \right)*\sum_{i=1}^{L}\alpha_{i}\delta \left(\tau -\tau_{i}\right)+r_{xn}\left(\tau \right) \end{array}$$

where $r_{xx}\left(\tau \right)$ is the cross-correlation result of the transmitted signal with itself, and $r_{xn}\left(\tau \right)$ is the cross-correlation result between the transmitted signal and the noise, and the convolution is represented by the ∗.

Rewriting it in vector form

(4) $$r_{xy}=r_{xg}\alpha +r_{xn}$$

where

$$\begin{array}{cc} r_{xy} & ={\left[r_{xy}\left(-N+1\right),r_{xy}\left(-N+2\right),\ldots ,r_{xy}\left(N-1\right)\right]}^{H}\\ r_{xn} & ={\left[r_{xn}\left(-N+1\right),r_{xn}\left(-N+2\right),\ldots ,r_{xn}\left(N-1\right)\right]}^{H}\\ r_{xg} & =[r_{xx_{1}}\left(-N+1\right),r_{xx_{2}}\left(-N+2\right),\ldots ,r_{xx_{M}}\left(N-1\right)]\\ r_{xx_{j}} & =[r_{xx}\left(-N+1-\tau_{j}\right),r_{xx}\left(-N+2-\tau_{j}\right),\\ \; & \ldots ,r_{xx}\left(N-1-\tau_{j}\right){]}^{H}\\ \alpha & ={\left[\alpha_{1},\alpha_{2},\ldots ,\alpha_{M}\right]}^{H}\in C^{K\times 1}\\ \; & r_{xy},r_{xn},r_{xx_{j}}\in C^{(2N-1)\times 1},r_{xg}\in C^{(2N-1)\times K} \end{array}$$

where $r_{xy}$ is the observation signal, representing the correlation result between the received signal and the transmitted signal, $r_{xg}$ denotes the dictionary matrix, which is used for the sparse representation of the observation signal, the *j*th column element of it is $r_{xx_{j}}$, $r_{xx_{j}}$ represents the correlation result between the transmitted signal and the transmitted signal with a time delay of *j*, $r_{xn}$ represents the correlation result between the noise and the transmitted signal, *K* is the number of atoms in the dictionary matrix, α represents the sparse vector, corresponding to the estimated values of the time delay and amplitude of the multipath signals.

The number of identifiable effective paths in a multipath channel is usually limited by the physical propagation mechanism, typically has sparse characteristics, and the sparsity degree *L* can be obtained through prior measurement. By applying a display constraint of ${∥\alpha ∥}_{0}\leq L$ and strictly following the physical sparsity characteristics of the signal path, the interpretability of the model and the efficiency of the algorithm can be improved. Therefore, the following optimization model is adopted in this paper

(5) $$\begin{array}{cc} \min_{\alpha } & {∥r_{xy}-r_{xg}\alpha ∥}_{2}^{2}\\ s.t. & {∥\alpha ∥}_{0}\leq L \end{array}$$

This model achieves efficient sparse recovery in scenarios with a known multipath order *L* by minimizing residual energy and rigally constraining sparsity [38].

## 3. Design of the Cross Correlation Oblique Projection MP Algorithm

In this section, we illustrate the drawbacks of the standard sparse recovery OMP/MP methods for the estimation of the delay of the multipath channel time domain and compare the CC-OPMP with OMP/MP methods to explain the relation between CC-OPMP algorithm and traditional sparse recovery. Then, details of the CC-OPMP algorithm are introduced.

**Figure 3:**
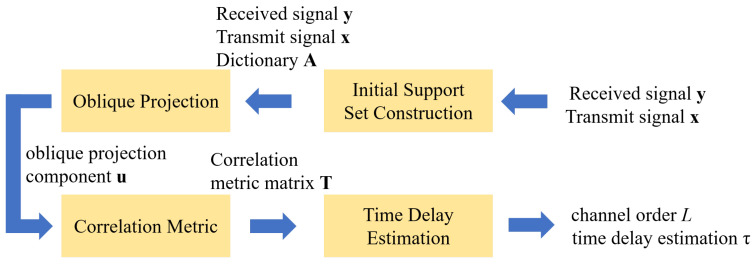
Algorithm framework.

Traditional OMP/MP algorithms operate under the mutually orthogonal channel assumption, recovering sparse signals via the model y=Ax. However, in practical multipath environments, strong inter-path correlations and noise interference induce time delay estimation deviations, which significantly degrade accuracy and limiting applicability in multipath scenarios. To overcome these limitations, this paper proposes the CC-OPMP algorithm. In model construction, a correlation-based post-processing framework enhances equivalent signal power while suppressing adverse low-SNR effects. In algorithm design, the sparse recovery framework and greedy atom selection principle are retained. Each path component of the received signal is projected into the orthogonal complement space corresponding to specific time delays, enabling effective multipath component separation and extraction. Concurrently, a correlation metric criterion is designed and implemented through a measurement matrix T. And multipath component count is determined by analyzing peak quantity in T, with peak locations extracted to estimate separated path delays.The algorithm framework is shown in the Figure 3.

To resolve temporal resolution limitations where single correlation peaks may encompass unresolved paths, we employ threshold-based peak detection to construct a constrained solution space for parameter estimation. This refined space is defined as

(6) $$\begin{array}{c} \Lambda =\left\{k\;|\;\sum_{n=0}^{N-1}y\left(n\right)x^{*}\left(n-k\right)>\lambda \right\},\;\mathrm{card}\left(\Lambda \right)=M \end{array}$$

where Λ represents the new support set, λ is the predefined threshold, *k* is an element within the support set Λ, and *M* denotes the number of elements in the support set. To ensure the detection of the main multipath components, we set λ to three times the mean value of the cross-correlation between the received signal *y* and the transmitted signal *x*.

The support set Λ obtained by the rough estimation of the correlation method constitutes a new dictionary matrix.

(7) $$\begin{array}{c} r_{xg}^{\Lambda }=\left[r_{xx}\left(k_{1}\right),\ldots ,r_{xx}\left(k_{m}\right),\ldots ,r_{xx}\left(k_{M}\right)\right]\in C^{2N-1\times M} \end{array}$$

Using oblique projection, the observation vector $r_{xy}$ is represented as

(8) $$\begin{array}{c} r_{xy}=r_{xg}^{\Lambda_{m}}\alpha_{m}+r_{xg}^{\Lambda_{-m}}\alpha_{-m}+r_{xn},m=1,2,\ldots M\; \end{array}$$

where $r_{xg}^{\Lambda_{m}}\in C^{2N-1\times 1}$ is the m-th basis vector in the dictionary matrix $r_{xg}^{\Lambda }$, and $r_{xg}^{\Lambda_{-m}}\in C^{2N-1\times M-1}$ is the matrix constructed by the rest vectors of the dictionary matrix $r_{xg}$ except the M-th basis vector.

To eliminate the impact of additional paths, oblique projection keeps the single path [39]. This makes it possible to obtain the orthogonal projection matrix $P^{⊥}\left(r_{xg}^{\Lambda_{-m}}\right)$ left multiplication Formula (7)

(9) $$\begin{array}{c} P^{⊥}\left(r_{xg}^{\Lambda_{-m}}\right)r_{xy}=P^{⊥}\left(r_{xg}^{\Lambda_{-m}}\right)r_{xg}^{\Lambda_{m}}+P^{⊥}\left(r_{xg}^{\Lambda_{-m}}\right)r_{xn}\; \end{array}$$

where,

(10) $$\begin{array}{cc} \; & P^{⊥}\left(r_{xg}^{\Lambda_{-m}}\right)r_{xg}^{\Lambda_{-m}}=O\\ \; & P^{⊥}\left(r_{xg}^{\Lambda_{-m}}\right)=\left[I-r_{xg}^{\Lambda_{-m}}{\left({\left(r_{xg}^{\Lambda_{-m}}\right)}^{H}r_{xg}^{\Lambda_{-m}}\right)}^{-1}{\left(r_{xg}^{\Lambda_{-m}}\right)}^{H}\right] \end{array}$$

After performing the dual-space decomposition operation, the signal vector is projected onto the complementary space. In terms of both theoretical and practical physical meanings, the signal vector will only produce a projection of the corresponding signal component in the complementary space when the correct path component exists within the complementary space. In other cases, the projection in the complementary space contains only noise components.

Therefore, based on the characteristic that there is a correlation between signals, while noise is uncorrelated with signals and with other noise, we adopt the maximum correlation criterion as the anti-interference correlation metric. By using the oblique projection vector $\mu_{m}=P^{⊥}\left(r_{xg}^{-m}\right)r_{xy}$ of the paths and the observed signal in the $r_{g}^{\Lambda }$ space, we construct the correlation metric matrix T as

(11) $$\begin{array}{c} T=\left[\begin{array}{cccc} T_{11} & T_{12} & \cdots & T_{1K}\\ T_{21} & T_{22} & \cdots & T_{2K}\\ \vdots & \vdots & \ddots & \vdots \\ T_{K1} & T_{K2} & \cdots & T_{KK} \end{array}\right] \end{array}$$

The correlation metric matrix $T\in R^{M\times M}$ quantifies the correlation measures among *M* oblique-projected vectors. During multipath signal processing, the residual signal after oblique projection requires further separation of valid signal components from noise. The core challenge lies in quantifying the correlation between atoms at different time delays and identifying physical multipath components. Traditional methods directly select atoms whose residual energy exceeds a detection threshold, but this approach becomes susceptible to noise interference under low SNR conditions.

Therefore, we design an evaluation matrix using the maximum correlation between signal components. The element $T_{\mathrm{ij}}$ at the *i*-th row and *j*-th column of matrix T is defined as

(12) $$\begin{array}{c} T_{\mathrm{ij}}=\max\left(\left|u_{i}*u_{j}^{H}\right|\right)\;i,j\in \Lambda \end{array}$$

where * represents convolution operation $T_{\mathrm{ij}}$ represents the correlation metric between the *i*-th and *j*-th atoms. When both atoms corresponding to *i* and *j* contain signal components, a local peak appears at position (i,j) of matrix T. Define the function findpeak(·) to search for local peaks. For a multipath channel order *L*, the position set Π of local peaks in T is

(13) $$\begin{array}{c} \Pi =\left(\begin{array}{cccc} \left(\Lambda_{1},\Lambda_{1}\right) & \left(\Lambda_{1},\Lambda_{2}\right) & \cdots & \left(\Lambda_{1},\Lambda_{L}\right)\\ \left(\Lambda_{2},\Lambda_{1}\right) & \left(\Lambda_{2},\Lambda_{2}\right) & \cdots & \left(\Lambda_{2},\Lambda_{L}\right)\\ \vdots & \vdots & \ddots & \vdots \\ \left(\Lambda_{L},\Lambda_{1}\right) & \left(\Lambda_{L},\Lambda_{2}\right) & \cdots & \left(\Lambda_{L},\Lambda_{L}\right) \end{array}\right) \end{array}$$

Since each true component generates *L* pairwise correlations, the target number of elements in Π is $L^{2}$. The number of true multipath components *L* is determined by analyzing the peak structure of T. Let *N* denote the total number of identified distinct peaks. The multipath channel order *L* satisfies

(14) $$\begin{array}{c} N=L^{2}\Rightarrow L=\left\lfloor \sqrt{N}\right\rfloor \end{array}$$

The number of peak elements in T is searched. Accurate estimation of the multipath channel order is achieved based on the statistical peak count, providing a priori information for subsequent multipath time delay estimation. Using this prior knowledge, each peak position is indexed through set Π. After removing duplicate elements in the set, the multi-path signal time delay estimates are obtained as

(15) $$\begin{array}{c} \hat{\tau }=\left\{\Lambda_{1},\Lambda_{2},\ldots ,\Lambda_{L}\right\} \end{array}$$

The CC-OPMP method suppresses noise by constructing a multipath delay estimation model in the correlation domain. During preprocessing, oblique projection isolates multipath components by projecting them onto orthogonal complement subspaces, reducing interference from strong path correlations. It combines subspace projection energy and inter-atom correlation to extract multipath information, suppressing spurious noise peaks and improving delay estimation accuracy. The proposed CC-OPMP is summarized as Algorithm 1.
**Algorithm 1** CC-OPMP Algorithm.1:**Input**   • Dictionary Matrix $r_{xg}^{\Lambda }$   • Observation vector $r_{xy}$2:**Output**   • Time Delay estimation $\hat{\tau }$3:**Initialization**   • Index set      $\Lambda =\{k|\sum_{n=0}^{N-1}y\left(n\right)s^{*}\left(n-k\right)>\lambda \}$, card(Λ) = *M*4:**Complementary space projection**5:   $\mu_{m}=P^{⊥}\left(r_{xg}^{\Lambda_{-m}}\right)r_{xy}$6:   $P^{⊥}\left(r_{xg}^{\Lambda_{-m}}\right)=\left[I-r_{xg}^{\Lambda_{-m}}{\left({\left(r_{xg}^{\Lambda_{-m}}\right)}^{H}r_{xg}^{\Lambda_{-m}}\right)}^{-1}{\left(r_{xg}^{\Lambda_{-m}}\right)}^{H}\right]$7:**Evaluation matrix construction**8:   $T=[T_{ij}]$, i,j∈Λ9:   $T_{\mathrm{ij}}=\max\left(\left|\mu_{i}*\mu_{j}^{H}\right|\right)$, i,j∈Λ10:**Peak detection**11:   $\Pi =findpeaks_{(i,j)\in \Lambda }T_{ij}$12:   $\hat{\tau }=\left\{\Lambda_{1},\Lambda_{2},\ldots ,\Lambda_{L}\right\}$

To further evaluate the computational complexity of the algorithm, the proposed CC-OPMP algorithm mainly includes four steps: cross-correlation, projection, construction of the metric matrix, and peak search. The computational complexity of each step is shown in Table 1.

Where *N* represents the number of sampling points of the received signal, *G* represents the size of the initial complete dictionary, *M* represents the size of the candidate set obtained from coarse estimation, *L* represents true sparsity of the channel.

In summary, the computational complexity of the CC-OPMP algorithm is $O(\left(2M+1\right)N\log N+M^{3}N+M^{4}+M^{2})$. Since N≫M, it can be simplified to $O(\left(2M+1\right)N\log N+M^{3}N)$. Compared with the computational complexity of the OMP algorithm, which is $O(LMN+L^{2}N)$, when the observation dimension *M* is small, the CC-OPMP algorithm has a computational complexity similar to that of OMP.

To further illustrate our conclusion, we will use a specific numerical example. Assume the following typical simulation parameters: N=3000, G=3000, L=5, M=30. We calculate the numerical complexity for the CC-OPMP algorithm. The results are shown in Table 2.

## 4. Simulation Result and Analysis

In this section, we compare the proposed CC-OPMP algorithm with the classical TDE method-matched filtering, OMP, SAMP, ROMP, Greedy-FISTA, and PCSBL. We analyze the algorithm performance from four aspects: multipath TDE results, the Mean Squared Error (MSE) of the algorithms, multipath channel order estimation results, and runtime. Based on the multipath TDE results and the MSE of each algorithm under different SNRs, we evaluate the estimation accuracy of the time delay parameter to reflect the estimation precision of the proposed algorithm. By comparing the runtime of each algorithm under the same conditions, we assess the computational efficiency of the proposed algorithm. Additionally, we calculate the accuracy of multipath channel order estimation by the proposed algorithm under different sparsity levels and SNRs to evaluate its stability.The definition of MSE is as follows:

(16) $$\begin{array}{cc} \; & \mathrm{MSE}=\frac{1}{K}\sum_{k=1}^{K}||y-\hat{y}|{|}_{2}^{2} \end{array}$$

where *K* represents the number of Monte Carlo simulations, y and $\hat{y}$ represents the origin and the reconstructing signals.

In this multipath signal transmission system, the transmitted signal employs 8PSK modulation with baseband processing at the receiver. The signal bandwidth is *B* = 20 kHz, sampling rate is $f_{s}$ = 120 kHz, sampling interval is $T_{s}=1/f_{s}$, pulse width is 20 ms, the noise is Gaussian white noise, and the number of Monte Carlo simulations is 1000.

### 4.1. Results of Multipath TDE

By comparing the multipath delay estimation results of different algorithms, the performance of the proposed CC-OPMP algorithm is evaluated. The number of multipaths *L* is set from 2 to 5, and the multipath delays are given by τ=[1000,1005,1002,1040,1044]Ts and multipath amplitude are given by α=[0.6+0.8j,0.5+0.6j,0.4+0.5j,0.4+0.4j,0.3+0.4j]. For each *L*, the first *L* values of τ are selected in order. The SNR is set to −5 dB and 0 dB, respectively.

**Figure 4:**
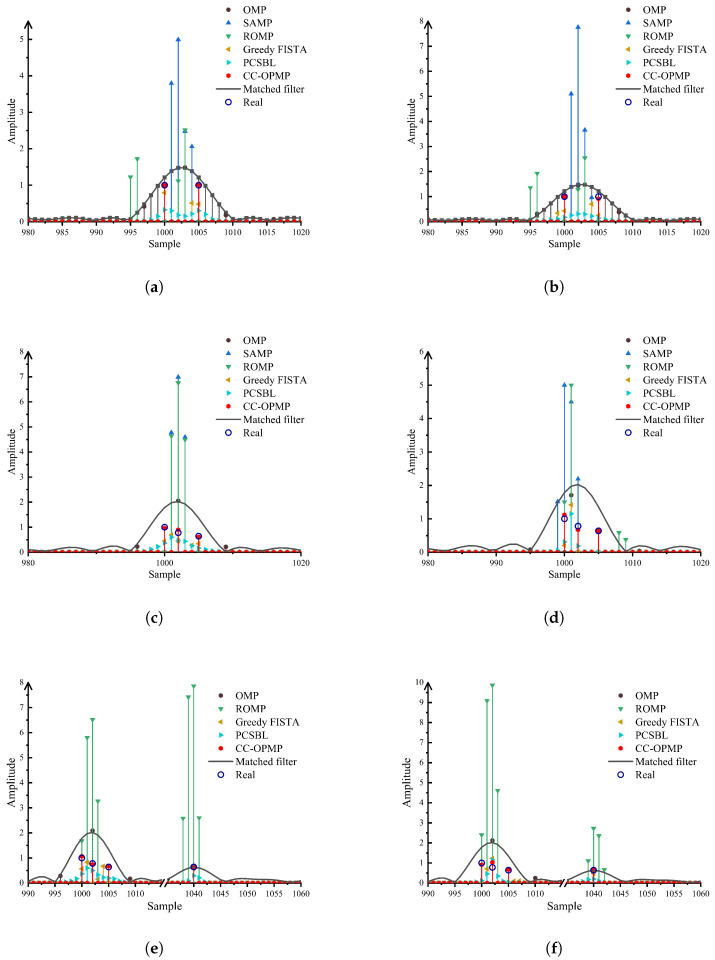

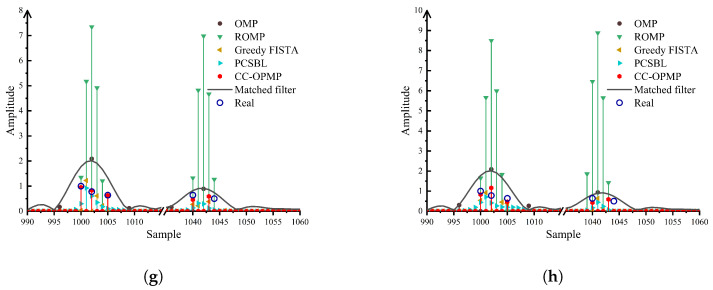
Multipath time delay estimation results of different algorithms. (**a**) 0 dB/L = 2. (**b**) −5 dB/L = 2. (**c**) 0 dB/L = 3. (**d**) −5 dB/L = 3. (**e**) 0 dB/L = 4. (**f**) −5 dB/L = 4. (**g**) 0 dB/L = 5. (**h**) −5 dB/L = 5.

The TDE results of each algorithm are shown in Figure 4. When the multipath number L is 2, the minimum delay interval is 5 sampling points. As shown in Figure 4a,b, the proposed CC-OPMP algorithm, the PCSBL algorithm, and the Greedy-FISTA algorithm accurately estimate the multipath delay. Due to the influence of multipath correlation, the delay estimation results of the OMP, SAMP, and ROMP algorithms are offset. When the multipath number L is 3 or 4, the minimum delay interval is 2 sampling points. As shown in Figure 4c,d, only the CC-OPMP algorithm accurately estimates the multipath delay. The other algorithms cannot accurately estimate the paths. When the multipath number L is 5, the minimum delay interval is 2 sampling points. The proposed CC-OPMP algorithm accurately estimates the first 4 paths, and there is a sampling point offset in the estimation result of the 5th path.

Compared to these algorithms, the proposed CC-OPMP algorithm overcomes the limitations of traditional greedy algorithms, achieving high-precision estimation of multipath time-delay parameters. Furthermore, it demonstrates higher time-delay resolution and parameter estimation accuracy compared to the PCSBL and Greedy-FISTA algorithms.

### 4.2. MSE Under Different SNRs

The SNRs are set from −20 dB to 30 dB with a 2 dB interval. The number of multipaths is L=3, and the multipath delays are $\tau =\left[1000,1002,1005\right]T_{s}$. By computing the MSE of multipath delay estimation under the above conditions for different algorithms, we compare the performance of the proposed CC-OPMP algorithm with other methods.

**Figure 5:**
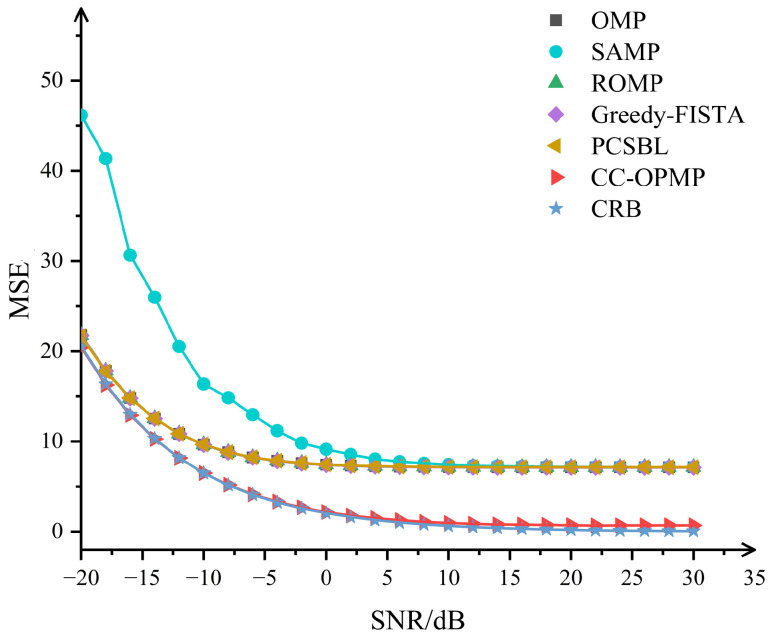
MSE in different SNR.

As shown in Figure 5, in the performance verification of multipath delay parameter estimation, the CC-OPMP algorithm demonstrates significant advantages. Within the SNR range of −20 to 30 dB, the MSE curve of CC-OPMP is at a relatively low level, outperforming the OMP, SAMP, ROMP, Greedy-FISTA, and PCSBL algorithms. The performance of each segment of the algorithm under different SNR is analyzed. When the SNR is low, the main factor affecting the algorithm performance is the noise level. The MSE of CC-OPMP is slightly lower than that of other algorithms, indicating that the CC-OPMP algorithm has certain advantages in the presence of strong noise. When the SNR ratio is high, the main factor affecting the algorithm performance is the strong correlation interference of paths. The CC-OPMP algorithm introduces the oblique projection technique to separate multipath, and the MSE of the CC-OPMP algorithm is significantly lower than that of other algorithms. This shows that the oblique projection technique has excellent performance in resisting multipath correlation interference. At the same time, the MSE performance of the CC-OPMP algorithm approaches the theoretical Cramer-Rao Lower Bound (CRLB). This indicates that the algorithm achieves the near-optimal estimation accuracy.

### 4.3. MSE Under Different SNRs and Sparsity Levels

In this section, the impacts of SNR and the sparsity levels on the performance of the algorithm are compared. We set the SNR to range from −10 dB to 30 dB with an interval of 2 dB, and the number of multipaths from 1 to 9. The multipath delay parameters are randomly generated, and the amplitude parameters attenuate as the delay increases.

**Figure 6:**
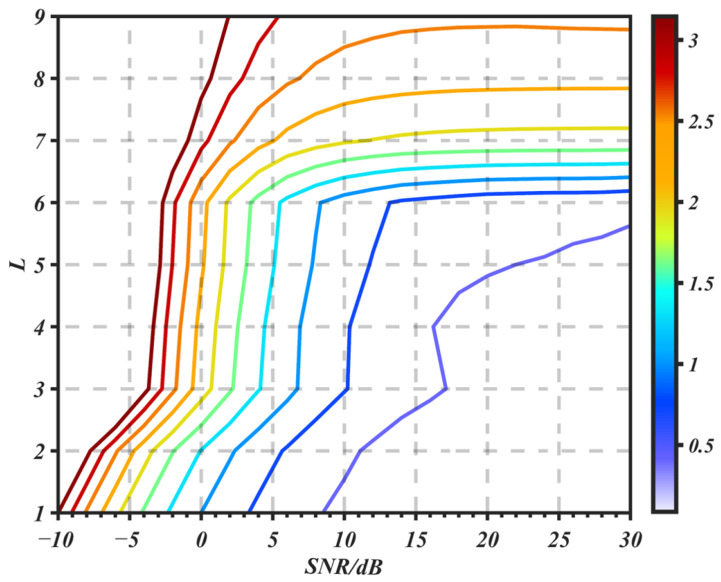
Effect of sparsity and SNR on estimation performance of the algorithm.

Figure 6 is a contour plot of the MSE. The same curve represents the settings of the number of multipaths and SNR corresponding to the same MSE. Curves of different colors correspond to different MSE values. The MSE is inversely proportional to the SNR and is directly proportional to the number of multipaths. In conclusion, when the SNR is greater than 0 dB and the number of multipaths is less than 6, the estimation accuracy of the CC-OPMP algorithm is excellent.

### 4.4. Estimation of Multipath Channel Order

**Figure 7:**
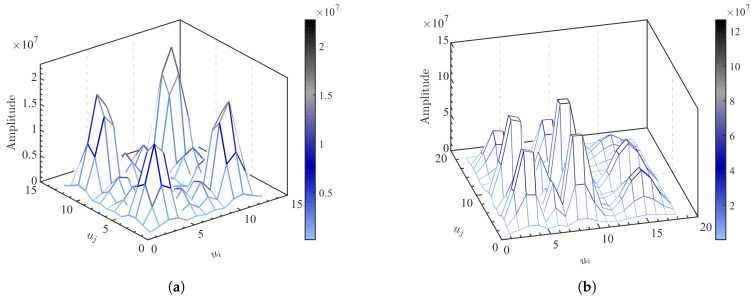
Correlation measurement matrix T. (**a**) 0 dB/L = 2. (**b**) 0 dB/L = 4.

When the SNR is low, the energy of the noise projected into each subspace will be greater than the signal energy. To enhance the algorithm’s robustness in low SNR condition, a criterion of maximum correlation is adopted by utilizing the correlation between signals and the non-correlation between noises. This criterion is used to extract the multipath components in each subspace after the oblique projection. The distribution of the correlation matrix T is shown in Figure 7. When the number of multipaths L is 2, there are signal projections in the two subspaces after the oblique projection. After correlation, there are four local peaks on the matrix T, as shown in Figure 7a. When the number of multipaths L is 4, there are signal projections in the four subspaces after the oblique projection. After correlation, there are 16 local peaks on the matrix T, as shown in Figure 7b. By counting the number of peaks, the channel order can be determined.

To verify CC-OPMP’s performance in multipath channel order estimation, we ran simulations with SNR from −10 dB to 20 dB in 5 dB steps, 6 multipaths, comparing it to Singular Value Decomposition (SVD)-based and Cross-Correlation (CC) methods.

**Figure 8:**
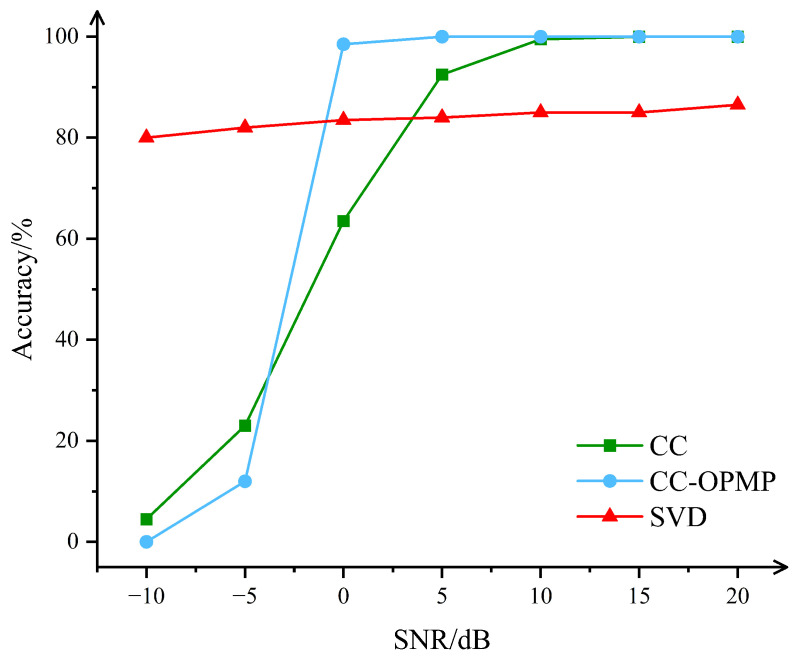
Effect of sparsity and SNR on estimation performance of the algorithm.

Figure 8 shows CC-OPMP excels at low SNR: at −10 dB, its correct rate is 80.0% and 4.5% for SVD and 0% for CC. But traditional methods surpass it at high SNR. This is as noise impact lessens, so CC-OPMP’s correlation-domain and oblique projection strategies for low-SNR robustness lose benefit. Also, oblique projection causes signal energy loss, limiting high-SNR accuracy and making it slightly worse than simpler traditional methods there. Still, since low-SNR scenarios are common in real ionospheric channels, CC-OPMP’s strong low-SNR performance makes it highly practical and reliable.

### 4.5. Algorithm Running Time

The computational efficiency of the proposed algorithm is verified. Table 3 compares the CPU running time of CC-OPMP with other algorithms at SNR = −5 dB. The result shows that the average running time of CC-OPMP algorithm is 0.0465 s, which is slightly higher than OMP/MP methods and significantly lower than PCSBL and Greedy-FISTA algorithm. Meanwhile, the CC-OPMP algorithm has better performance compared with the above algorithms.

## 5. Conclusions

This paper addresses the challenge of super-resolution multipath delay estimation in ionospheric channel measurement and proposes a high-resolution multipath delay estimation algorithm named CC-OPMP. In practical environments with low SNR and highly correlated multipath signals, traditional greedy algorithms such as matching pursuit exhibit significant performance degradation. The core contribution of the CC-OPMP algorithm lies in its proposal of a novel framework that combines a dual-space decomposition mechanism, oblique projection technology, and correlation domain processing. The algorithm first decomposes the signal space into a signal subspace and its orthogonal complement space, then uses oblique projection technology to accurately project multipath components onto their respective subspaces, achieving effective separation of strongly correlated multipath signals and interference suppression. Processing in the correlation domain not only improves the output SNR but also significantly suppresses noise interference. Ultimately, while maintaining a computational complexity comparable to that of the classic OMP algorithm, CC-OPMP substantially enhances delay estimation accuracy and anti-interference performance under low SNR and strong correlation channel conditions. Simulation results demonstrate that the proposed algorithm outperforms traditional methods in terms of delay resolution, providing an effective solution for high-precision delay estimation in ionospheric channels. In future work, we will focus on accounting for the effects of Doppler shift and off-grid delays, and further explore the algorithm’s potential in ionospheric time-varying channel models and practical system integration.

## Figures and Tables

**Table 1:** Computational complexity of each step.

Cross-Correlation	Projection	Construction of Metric Matrix	Peak Search
O((M+1)NlogN)	$O(M^{3}N+M^{4})$	O(MNlogN)	$O(M^{2})$

**Table 2:** Analysis of numerical complexity of OMP and CC-OPMP algorithm.

Algorithm	Asymptotical Complexity	Numerical Complexity
OMP	$O(LGN+L^{2}N)$	$4.507\;\times \;10^{7}$
CC-OPMP	$O(\left(2M+1\right)N\log N+NM^{3}+M^{4}+M^{2})$	$8.392\;\times \;10^{7}$

**Table 3:** Average CPU running time comparison of algorithms (Unit: seconds).

Algorithm	Running Time	Algorithm	Running Time
CC	0.00007	CoSaMP	0.0053
OMP	0.02889	Greedy-FISTA	32.9351
SAMP	0.01450	PCSBL	76.9411
ROMP	0.02523	CC-OPMP	0.0465

## Data Availability

The original contributions presented in this study are included in the article. Further inquiries can be directed to the corresponding author.
